# Mechanism of Yangxinshi Intervention on Cardiac Fibrosis in Diabetic Cardiomyopathy Based on Network Pharmacology

**DOI:** 10.1155/2022/3968494

**Published:** 2022-01-21

**Authors:** Jiangying Kuang, Kaiyi Wu, Wenjing Li, Xuguang Zhang, Hao Zhang, Zhiyi Jia, Qingmei Han, Xiaochen Tian, Rong Sun, Qinghua Lu, Yusheng Liu

**Affiliations:** ^1^Department of Cardiology, The Second Hospital, Cheeloo College of Medicine, Shandong University, Jinan, Shandong, China; ^2^Tianjin University of Traditional Chinese Medicine (TCM), Tianjin, China; ^3^Department of Cardiology, The Fourth People's Hospital of Jinan, Jinan, China; ^4^Basic Medical Research Institute, The Second Hospital, Cheeloo College of Medicine, Shandong University, Jinan, Shandong, China

## Abstract

**Background:**

Cardiac fibrosis (CF) is major myocardial change in diabetic cardiomyopathy (DCM). Yangxinshi as a Chinese medicine formula is used to treat cardiovascular diseases. However, the exact effective mechanism of Yangxinshi on CF is still uncertain. Hence, based on the pharmacological network, predicting the active components, potential targets and pathways of Yangxinshi on diabetic fibrosis require to be further studied.

**Materials and Methods:**

By using Cytoscape 3.6.0 Bisogenet plug-in, the active components of Yangxinshi were obtained and screened through TCMSP, and the PPI network of DCM-CF was constructed and then screened by CytoNCA plug-in. GO analysis and KEGG pathway enrichment analysis were carried out by Cluego plug-in. Combined with the results of network pharmacological analysis, cells *in vitro* were performed to verify the CF stimulated with high glucose or intervence with Yangxinshi, and the expressions of Cbl-b, *p*-smad2, and *α*-SMA were detected.

**Results:**

Yangxinshi might play a key role in reversing cardiac fibrosis in individuals with DCM by regulating the signal pathway of CBL and promoted the expression of Cbl-b and inhibited the expression of *p*-smad2 and *α*-SMA, verifying some predictive work *via network* pharmacology.

**Conclusion:**

Based on *network* pharmacology, this study demonstrates that the beneficial effect of Yangxinshi on CF is related to the Cbl-b/smad2 *pathway*, providing an idea for the therapeutic effect of Yangxinshi on cardiac fibrosis in DCM.

## 1. Introduction

Diabetic cardiomyopathy (DCM), as a common complication of diabetes, is a major risk factor for heart failure [1, 2]. DCM is a disease that leads to cardiac remodeling due to multiple effects of oxidative stress, energy metabolism disorder, hyperglycemia, insulin resistance, inflammation, and so on [3–5]. Cardiac fibrosis (CF) is the major pathological change of cardiac remodeling leading to abnormal cardiac structure and cardiac function [6, 7]. However, no effective methods for the treatment of CF have been found. Searching potential drugs for the treatment of CF is important in DCM.

Casitas B lymphoma-b (Cbl-b), a RING finger E3 ubiquitin ligase, has been identified as a critical regulator of adaptive immune responses and is potential therapeutic targets for treating obesity-induced insulin resistance [8] and insulin-dependent diabetes mellitus [9]. Recent study has demonstrated that Cbl-b is closely related to liver fibrosis and inflammation [10]. Gruber found that Cbl-b might control TGF-*β* signaling by directly targeting Smad-7. TGF-*β* plays a key role in fibrosis by regulating the phosphorylation and nuclear translocation of Smad2/3. *α*-SMA is a fibrotic marker and is also regulated by TGF-b [11].

Network pharmacology is a systematic analysis method which aims at analyzing the intervention of multimolecular drugs on disease network and constructing drug composition-gene-target-disease network to reveal the pharmacological mechanism of multimolecular drugs for traditional Chinese medicine [12].

Yangxinshi is composed of thirteen kinds of Chinese herbal medicine, such as ginseng, *Rhizoma corydalis*, *Codonopsis pilosula*, *Pueraria lobata*, hawthorn, and so on. According to research, Yangxinshi can improve cardiac function and reduce CF and reverse ventricular remodeling by regulating oxidative stress, improving energy metabolism, reducing inflammation, protecting endothelial function, and so on [13–16].^.^Hence, we speculate that Yangxinshi may have beneficial effect on the pathological process of CF in DCM.

In this study, we combined experiments *in vitro* with network pharmacology to explore the characteristics of Yangxinshi and revealed the potential effect of it on CF *via* Cbl-b/Smad signal pathway in DCM, providing ideas for the future research of traditional Chinese medicine for the treatment of CF in DCM.

## 2. Materials and Methods

### 2.1. Screening of Drug Ingredient Information of Yangxinshi

The chemical constituents of 13 kinds of herbs (*Ganoderma lucidum*, *Salvia miltiorrhiza*, *Rhizoma corydalis*, *Angelica sinensis*, *Radix rehmanniae*, *Herba epimedii*, *Coptis chinensis*, *Pueraria lobata*, hawthorn, *Codonopsis pilosula*, *Radix astragali*, ginseng, and *Radix glycyrrhizae*) in Yangxinshi were searched using the Traditional Chinese Medicine Systems Pharmacology Database and Analysis Platform (TCMSP) (https://tcmspw.com/tcmsp.php), Traditional Chinese Medicine Integrative Database (TCMID), (http://www.megabionet.org/tcmid), Chinese Ethnic Minority Traditional Drug Database (CEMTDD) (http://www.cemtdd.com/index.html), and Yet Another Traditional Chinese Medicine Database (*Ya TCM*) (http://cadd.pharmacy.nankai.edu.cn/yatcm/home). Based on pharmacokinetic parameters, oral bioavailability (OB) ≥ 30% and drug likeness (DL) ≥ 0.18 were used as limiting conditions to screen and collect chemical constituents.

### 2.2. Construction and Analysis of the Yangxinshi-Component-Target Network

The protein targets of Yangxinshi's drug components were predicted and collected according to the TCMSP database. Combined with UniProKBt search function of the UniProt database (https://www.uniprot.org/), the name of target gene was corrected to its official name which the species was defined as “*human*”. Constructed Yangxinshi-Component-Target network by integrating the active ingredient and target information of Yangxinshi, importing the *network* visualization software Cytoscape 3.6.0.

### 2.3. Construction of Protein-Protein Interaction (PPI) Network between CF and DCM

According to databases of GeneCards (https://www.genecards.org/), CTD (http://ctdbase.org/), and PubMed (https://www.ncbi.nlm.nih.gov/pubmed), using “*diabetic cardiomyopathy*” and “*cardiac fibrosis*” as keywords, the disease-related targets were searched and screened and the related targets were collected eventually. The target information collected by each database is integrated and de-duplicated to obtain the final related targets of DCM and CF. The targets are introduced into the network visualization software Cytoscape 3.6.0, and the Bisogenet plug-in is used to construct the protein–protein interaction (PPI) network [17] of DCM and CF. Through the merge function of the software, the intersection network of DCM and CF is obtained. It is predicted to be the target network of CF in DCM. The whole article has been screened for three times, and network pharmacology must undergo node degree screening, from the first ① oral utilization absorption and drug type screening of compounds, ② PPI network characteristic data are screened according to the median, and ③ the final PATHWAY and GO analysis are screened by *p*-value, leaving the display with the smallest *p*-value.

### 2.4. Construction of PPI Network about the Target of CF in DCM Treated by Yangxinshi

The component-target PPI network was constructed using the network visualization software Cytoscape 3.6.0. Through the function of the software Merge, target PPI network intersection of Yangxinshi target and CF in DCM target was obtained, and target regulatory network of Yangxinshi intervention mechanism of diabetic myocardial fibrosis was obtained. The CytoNCA plug-in is used to filter parameters of the network, such as degree, eigenvector, betweenness, network, local average connectivity (LAC), closeness. For the first time, on the condition that the values of degree, eigenvector, betweenness, network, LAC and closeness are greater than or equal to twice the median. In the second screening, the PPI network of the core target is obtained under the condition that it is greater than or equal to the median of its degree value. The genes in the core target PPI network are derived, corrected name by Uniprot database, and then analyzed in the next step.

### 2.5. GO Analysis and KEGG Pathway Enrichment Analysis

Use Cytoscape 3.6.0 plug - in Cluego for analysis. Through GO analysis and KEGG pathway analysis of the key targets, the potential mechanism of Yangxinshi on CF in DCM was obtained.

### 2.6. Cells, Yangxinshi, and Reagents

The primary cardiac fibroblasts of neonatal rats (purchased from the Experimental Animal Center of Shandong University) were extracted and cultured in complete DMEM (low glucose) medium (10% fetal bovine serum, 1% penicillin - streptomycin) and were cultured at 37 °C with 5% CO_2_. The cells in logarithmic growth phase were taken for experiment.

Yangxinshi powder (provided by *Qingdao Guofeng Pharmaceutical*) was dissolved in DMEM (low glucose) medium in 5 g/L, bathed in water at 37°C for 30 min, dissolved by ultrasound for 30 min at 3000 rpm/min centrifugation for 10 min, and filtrated sterilization.

The CCK-8 cell proliferation detection kit (batch number: 042419190 821) was purchased from *Hangzhou Biyuntian Biological Biology*, *β*-tublin antibody (AC021) was purchased from *AB clonal company*, Cbl-b antibody (12781-1-AP) was purchased from *Proteintech company*, p-smad2 antibody (3108) was purchased from *Cell signaling company*, and *α*-SMA antibody (19245) was purchased from *Cell signaling company*.

### 2.7. Detection of the Viability of Rat Cardiac Fibroblasts by CCK - 8

Cardiac fibroblasts in the logarithmic growth phase were inoculated in 96-well plates with a density of 9000 cells per well in DMEM low-glucose complete medium. The cells were cultured at 37 °C with 5% CO_2_ for 24 hours and then added Yangxinshi at the concentrations of 0, 0.00005, 0.0005, 0.005, 0.005, 0.0005, and 0.5 g/L. After 24 hours, the culture medium was abandoned and 100 *μ*l 5% CCK- 8 solution was added to each well. Put it into the incubator and continue to culture for 1 hour. The absorbance at 450 nm was measured by an enzyme labeling instrument, and the cell survival rate was calculated according to the following formula: cell viability (%) = (mean absorbance value of experimental group-absorbance value of blank group)/(mean absorbance value of control group-absorbance value of blank group) × 100%.

### 2.8. Cell Grouping

The cultured cardiac fibroblasts were divided into the low glucose control group, low glucose + Yangxinshi group, high glucose group, and high glucose + Yangxinshi group. The low glucose group was treated with 5.5 mmol/L glucose medium, the high glucose group was treated with 50 mmol/L glucose medium, the control group was not treated with Yangxinshi, and the Yangxinshi group was treated with 0.05 g/L Yangxinshi.

### 2.9. Western Blot

The cardiac fibroblasts of each group were collected and washed with phosphate buffer solution (PBS) for 3 times. The total protein was extracted and heated at 99°C for 10 min. According to the relative molecular weight of the protein, sodium salt–polyacrylamide gel electrophoresis (SDS-PAGE) was performed. The protein was transferred to PVDF membrane at constant current. The membranes were then incubated with primary antibodies overnight, including Cbl-b, *α*- SMA, *p*-smad2, and *β*- Tublin. Following incubation with the appropriate secondary antibodies for 1.5 hours, the membranes were visualized by chemiluminescence using ECL detection reagents.

### 2.10. Statistical Analysis

SPSS 22.0 statistical software (SPSS Inc., Chicago, IL, USA) was used to analyze the experimental data. The experimental data were expressed as mean ± S.E.M. One-way analysis of variance (ANOVA) was used to compare the groups, and LSD test was used for pairwise comparison between groups, *p* value < 0.05 was considered to be statistically significant. The *p*-value set for the enrichment analysis also was ≤0.05.

## 3. Results

### 3.1. Screening of Active Components of Each Single Drug Compound

**Figure 1:**
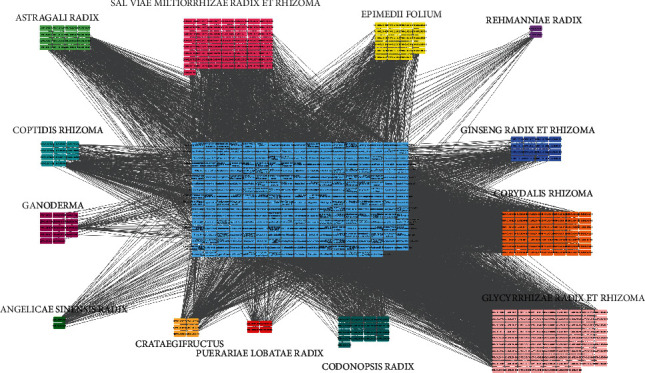
Network of “yangxinshi-ingredients-prediction target”.

Using TCMSP database and related literature, the main active components of Yangxinshi were excavated and collected and screened by OB and DL values. The results showed that the active components of Yangxinshi were 65 of *Salvia miltiorrhiza*, 23 of *Herba epimedii*, 2 of *Radix rehmanniae*, 14 of *Coptis chinensis*, 61 of *Ganoderma lucidum*, 92 of *Radix glycyrrhizae*, 20 of *Radix astragali*, 22 of ginseng, 59 of *Rhizoma corydalis*, 2 of *Angelica sinensis*, 6 of hawthorn, 4 of *Pueraria lobata*, and 21 of *Codonopsis pilosula*. The composition and target information were imported into Cytoscape 3.6.0 software to construct the ingredient-target network (Figure 1).

### 3.2. Prediction of Targets for DCM and CF

**Figure 2:**
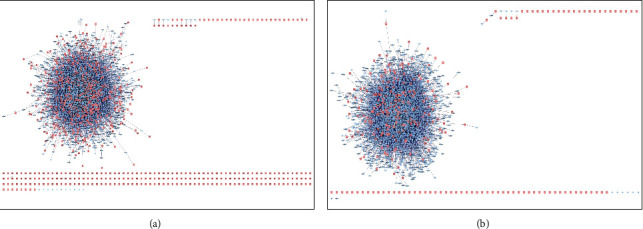
PPI network of DCM targets (a) and CF targets (b).

Total of 904 targets were found in GeneCards, PubMed, and CTD databases with “diabetic cardiomyopathy” as the keyword, and total of 340 targets were found in GeneCards, PubMed, and CTD databases with “cardiac fibrosis” as the keyword. The related targets were introduced into the Cytoscape 3.6.0 software. By constructing the disease PPI network of the DCM-related targets, a network with total of 4639 direct or indirect targets and 28462 interrelationships were obtained. By constructing the disease PPI network of the CF-related targets, a network with total of 3440 direct or indirect targets and 20753 interrelationships were obtained (Figures 2(a) and 2(b)).

### 3.3. Construction of PPI Network of DCM-CF Treated with Yangxinshi

**Figure 3:**
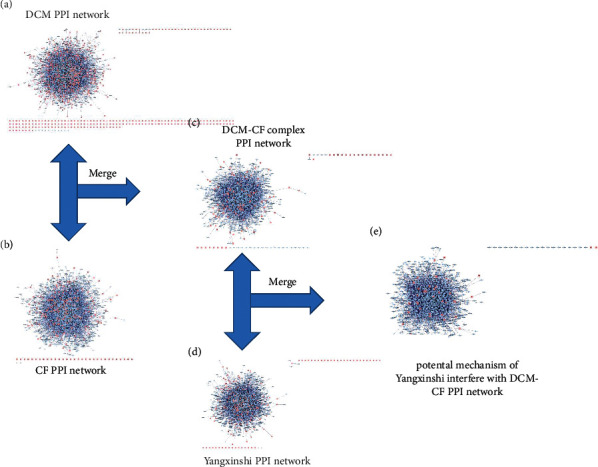
Potential mechanism of Yangxinshi interfere with DCM-CF PPI network. (a) DCM PPI network. (b) CF PPI network. (c) DCM-CF complex PPI network. (d) Yangxinshi PPI network. (e) Potential mechanism of Yangxinshi interfere with DCM-CF PPI network.

Constructed the component PPI network of the active components of Yangxinshi using the BisoGenet plug-in in Cytoscape 3.6.0 software. A network with total of 2907 direct or indirect targets were obtained, and the interactions between the targets was 17920. The component-target-disease interaction PPI network is constructed using the merge function of Cytoscape 3.6.0 software, Yangxinshi component interferes with the potential mechanism of DCM - CF PPI network. A PPI network with total of 1483 targets and 7308 interactions between targets of DCM and CF was obtained. Data are shown (Figures 3(a)–3(e)).

### 3.4. Screening Targets of Yangxinshi Interfere with DCM-CF PPI Network

Select Yangxinshi interfere with DCM-CF PPI network, analyze the network, and use Cytoscape 3.6.0 software to analyze the PPI network data. Screening targets through the Cyto NCA plug-in in Cytoscape 3.6.0 software, with the limitation of degree, eigenvector, betweenness, network, LAC, closeness indicator. First, the nodes larger than the median of each index were screened, and then the nodes greater than the median degree value were used to obtain the key targets of Yangxinshi interfere with DCM-CF PPI network. The results showed that Yangxinshi mainly acted on TAK1- binding protein- 1 (TAB1), focal adhesion kinase-1 (FAK1), and hemato poietic cell kinase (HCK), serine/threonine kinase 24 (STK 24), DEAD-box protein5 (DDX5), and other key targets. The results of the first 20° values of key targets are shown in Table 1.

**Figure 4:**
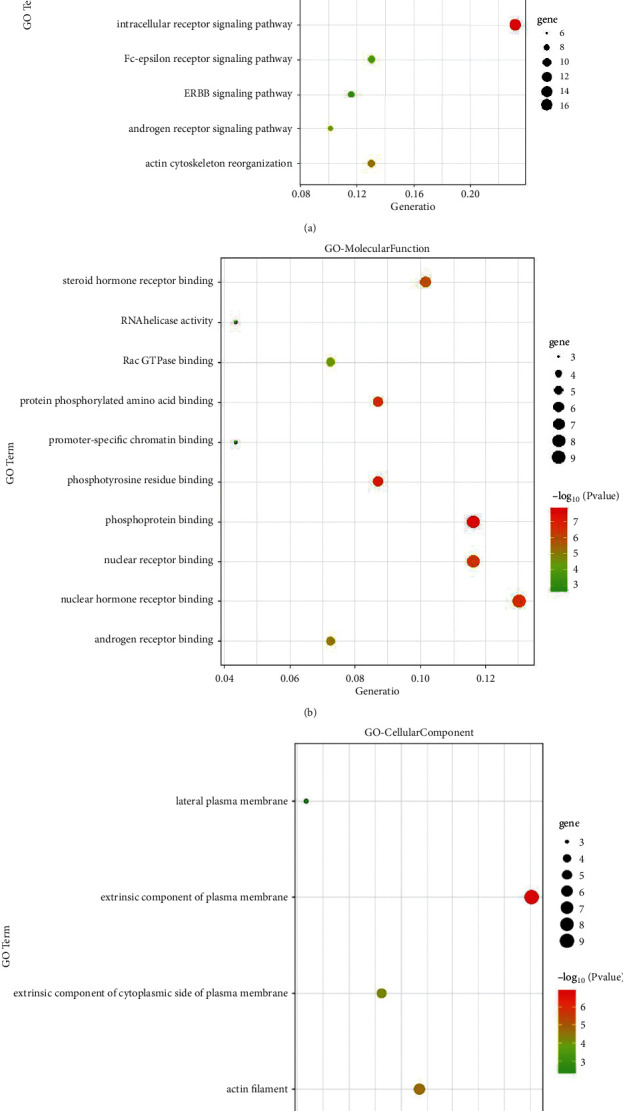
GO analysis of key targets. (a) Biological process, (b) molecular function, and (c) cells component.

**Figure 5:**
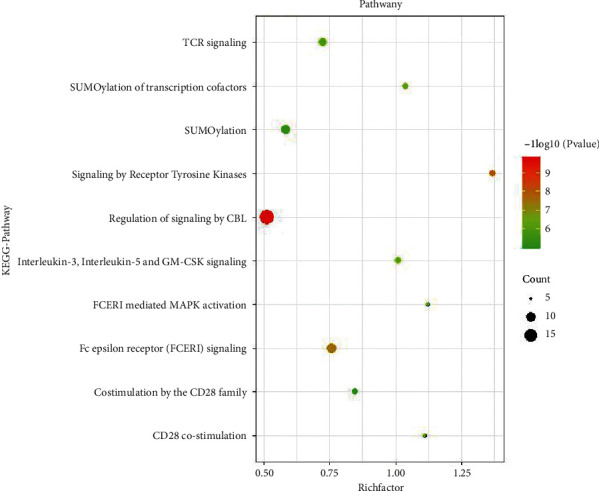
KEGG pathway enrichment analysis.

The targets were analyzed by GO analysis and KEGG pathway enrichment analysis, in which GO analysis included biological process, molecular function, and cell composition. The results showed that Yangxinshi mainly through biological processes, such as intracellular receptor signaling pathway, intracellular steroid hormone receptor signaling pathway, and positive regulation of apoptosis signaling pathway; molecular functions, such as nuclear hormone receptor binding, phosphoprotein binding, and nuclear receptor binding; cellular component, such as extrinsic component of plasma membrane and actin filament; and key pathways, such as regulation of signaling by CBL, CD28 co-stimulation, FCERI-mediated MAPK activation, and other key pathways, to play the role of reversing CF in DCM (Figures 4 and 5).

### 3.5. The Suitable Concentration of Yangxinshi Effect on Cardiac Fibroblasts

After the primary cardiac fibroblasts were treated with Yangxinshi (0, 0.00005, 0.0005, 0.005, 0.005, 0.00005, 0.5 g/L) for 24 hours, the CCK-8 results showed that there was no obvious toxic effect on the cells at or below 0.05 g/L, but at the concentration of 0.5 g/L, the cytotoxic effect was significant, and the difference was statistically significant (based on the concentration of 0 g/L). (F _high glucose group_ = 35.9, *P*_high glucose group_ < 0.0001. F_low glucose group_ = 57.4, *p* < 0.0001). According to the effect of Yangxinshi on the activity of primary cardiac fibroblasts, 0.05 g/L was selected as the intervention concentration. The results are shown in Table 2.

### 3.6. Western Blot

**Figure 6:**
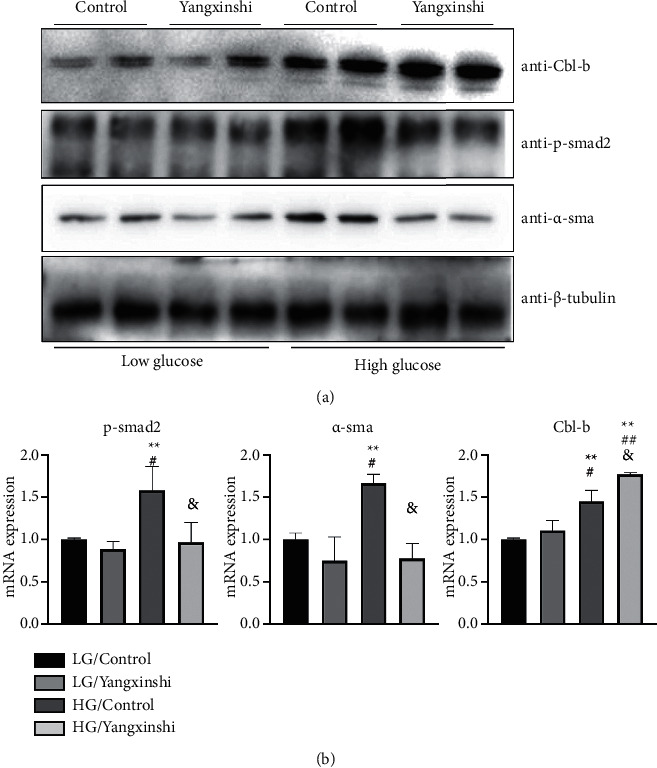
Yangxinshi inhibits the p-samd2, *α*-sma, and Cbl-b genes expression. (a) Western Blot analysis of p-samd2, *α*-sma, and Cbl-b genes' expression treated with low glucose (5.5 mM) or high glucose (50 mM) and with or without Yangxinshi (each group has three samples, and each experiment was repeated three times). (b) Quantitative results of Western blot in.

The results of western blot showed that the expressions of Cbl-b, *p* - smad2, and *α*- SMA in the high glucose group were significantly higher than those in the low glucose group and the low glucose + Yangxinshi group (*P*_Cbl-b low glucose control group_ = 0.008, *P*_p-smad2 low glucose control group_ = 0.038, *P*_*α*-SMA low glucose control group_ = 0.02). Compared with the high glucose group, the expression of Cbl-b in the high glucose + Yangxinshi group increased significantly, and the difference was statistically significant (*P*_Cbl-b low glucose control group_ = 0.001, *P*_Cbl-b low glucose + Yangxinshi group_ = 0.002, *P*_Cbl-b high glucose control group_ = 0.023), the expression of p-smad2 and *α*-SMA decreased significantly (*P*_p-smad2 high glucose control group_ = 0.032, *P*_*α*-SMAglucose control group_ = 0.008). The results are shown in Figure 6.

## 4. Discussion

Diabetes is a chronic disease that seriously endangers the human body, which can damage to the structure and function of many organs, such as the heart, the kidney, and the brain. Diabetic cardiomyopathy (DCM) is closely related to heart failure, especially diastolic dysfunction [18]. Myocardial fibrosis is one of the characteristics of diabetic cardiomyopathy, which is the focus of DCM [19]. Under the stimulation of high glucose environment, cardiac fibroblasts are activated into myofibroblasts, secreting a large amount of collagens, and extracellular matrix expands, thus promoting the occurrence of CF in DCM [20–22]. Hence, to avoid the development of diastolic heart failure [23, 24] is to prevent and treat the pathological changes of CF in diabetic individuals.

Previous data [25–27] suggested that Yangxinshi was beneficial for the treatment of coronary heart disease, arrhythmia, heart failure, hypertension, and diabetes. Meanwhile, Yangxinshi could regulate oxidative stress, improve energy metabolism, reduce inflammation, and protect the endothelial function. Therefore, we suppose that Yangxinshi has a certain preventive and therapeutic effect on CF in DCM. In this research, based on the characteristics of multicomponents, multitargets, and multipathways of proprietary the Chinese medicine, the PPI network of Yangxinshi in the treatment of CF in DCM was constructed by network pharmacology. The key components, targets, and pathways of Yangxinshi in the treatment of CF in DCM were screened, and the main pathways were further verified by cell experiments in vitro.

Based on the network pharmacology, TAB1, FAK1, HCK, STK24, DDX5, and more were regarded as the core targets of treatment of CF in DCM with Yangxinshi. TAB1 is the binding protein of TGF-*β* activated kinase-1 (TAK-1). TAK1 is activated by transforming growth factor *β* (TGF- *β*) to form a complex with TAB1, regulating the extracellular matrix, and promoting the occurrence of CF [28–30]. FAK1 is a nonreceptor protein tyrosine kinase, which plays an important role in regulating the migration, proliferation, and survival of many kinds of cells [31, 32]. Published data [33, 34] showed that inhibiting the expression of FAK1 could downregulate *α*- SMA, by regulating TGF- *β* and playing a protective role in the pathological process of CF. HCK as a member of sarcoma family of tyrosine kinases (SFKs) may activate mitogen-activated protein kinase (MAPK) pathway, leading to the activation of NF- *κ*B, and might reverse cardiac fibrosis in DCM. STK24 as a member of the mammalian Sre20 kinase family can improve the insulin resistance by inhibiting the insulin signaling pathway, as we all know that insulin resistance is one of the key factors of myocardial fibrosis in DCM [35]. DDX5, a member of the death box protein family, is an ATP- dependent RNA helicase, which may interfere with the occurrence and development CF in DCM via affecting the activity of NF-*κ*B [36, 37].

By the GO analysis and KEGG pathway enrichment analysis, the preventive effects of Yangxinshi on CF in DCM were complex, versatile characteristics of multitargets and multipathways, mainly through biological processes such as intracellular receptor signaling pathway, intracellular steroid hormone receptor signaling pathway, key pathways such as regulation of signaling by CBL, CD28 co-stimulation, FCERI-mediated MAPK activation, among which CBL pathway has the highest weight. CBL family proteins are a class of E3 ubiquitin ligases containing RING finger domain, including c- Cbl, Cbl-b, and Cbl-c [38, 39]. Cbl associated protein (CAP) is a downstream signal molecule of glucose transporter 4 (GLUT4) in glucose metabolism, and its CAP/CBL pathway is one of the insulin signal pathways, which participates in glucose uptake by cells [40, 41].

Several studies [9, 42] have confirmed that the deletion of Cbl-b in the CBL family is closely related to the occurrence of diabetes. Targeted therapy of Cbl-b might be used as a potential strategy to reduce the insulin resistance in diabetes and then inhibit the progression of myocardial fibrosis in diabetic cardiomyopathy. In this research, we screened out the best drug concentration of Yangxinshi was 0.05 g/L by CCK - 8 method in order not to have toxic effect and verified that Yangxinshi could inhibit increasing the numbers of primary myocardial fibroblasts under the high glucose 50 mmol/L glucose environment by the prediction of network pharmacology. TGF - ß/Smads pathway is an important pathway in the occurrence and development of CF [43]. Western blot showed that the expression of *p*-smad2 and *α* - SMA in the high glucose control group was significantly higher than that in the low glucose group. In the high glucose group, the expression of *p*-smad2 and *α*- SMA increased, which verified the inducing effect of high glucose on CF. After the treatment of Yangxinshi, the expression of *p*-smad2 and *α*- SMA was significantly decreased, and the expression of Cbl-b was significantly increased in the high glucose with Yangxinshi treatment group.

According to the experimental results in vitro, we speculate that Yangxinshi has a therapeutic effect on CF in DCM by regulating CBL pathway, promoting the expression of Cbl-b and inhibiting TGF- *β*/Smads pathway. This conclusion is consistent with the results of network pharmacological analysis. This study verified that Yangxinshi's role in reversing CF in DCM by regulating CBL pathway by analyzing network pharmacology of selecting the key biological mechanism about the intervention of Yangxinshi. However, the mechanism of CF in DCM is complex, and the exact mechanism of effects of Yangxinshi on CF in DCM still requires to be further explored in vivo and in vitro.

In summary, the effect of Yangxinshi on cardiac fibrosis in DCM is revealed, probably by regulating CBL pathway, providing a new train of thoughtful and theoretical basis for further research on the mechanism of pathological changes.

## Figures and Tables

**Table 1:** Key targets of Yangxinshi interfere with DCM-CF PPI network, cardiac fibrosis PPI network, GO analysis, and KEGG pathway enrichment analysis.

No.	Target name	Degree value	Node tightness	Node dielectric
1	TAB1	10	0.24137931	0.32152115
2	FAK1	9	0.23106796	0.23090253
3	HCK	7	0.24236253	0.19389688
4	STK24	6	0.22284644	0.27417747
5	DDX5	6	0.18652038	0.11337416
6	TYY1	6	0.21557971	0.17403979
7	PAK1	6	0.20411664	0.06395813
8	CDC37	5	0.20135364	0.11921379
9	CSK	5	0.14302885	0.06637231
10	ERBB4	5	0.21099291	0.04459479
11	NUCL	5	0.1973466	0.04985045
12	SH3G3	5	0.19381107	0.11251958
13	CRKL	5	0.238	0.1955823
14	NCK2	5	0.19572368	0.01532308
15	YES	5	0.24089069	0.2605042
16	2AAA	5	0.18506998	0.06622988
17	PDC6I	5	0.26327434	0.48871481
18	LCP2	5	0.20446735	0.06544652
19	DVL2	5	0.19572368	0.03347102
20	STK4	4	0.12646121	0.12647771

**Table 2:** Effect of Yangxinshi on cardiac fibroblasts viability.

	Concentration (g/L)	Cell viability low glucose group (%)	Cell viability high glucose group (%)
Yangxinshi	0	100 ± 7	100 ± 6
	0.00005	90 ± 13	103 ± 3
	0.0005	92 ± 7	104 ± 13
	0.005	102 ± 3	118 ± 6
	0.05	132 ± 11	130 ± 9
	0.5	70 ± 11^∗^	61 ± 12^∗^

## Data Availability

The data used to support the findings of this study are available from the corresponding author upon request.
